# Revised Pediatric Reference Data for the Lateral Distal Femur Measured by Hologic Discovery/Delphi Dual Energy X-Ray Absorptiometry

**DOI:** 10.1016/j.jocd.2009.01.005

**Published:** 2009-03-24

**Authors:** Babette S. Zemel, Virginia A. Stallings, Mary B. Leonard, Donna R. Paulhamus, Heidi H. Kecskemethy, H. Theodore Harcke, Richard C Henderson

**Affiliations:** 1 Division of Gastroenterology, Hepatology and Nutrition, The Children's Hospital of Philadelphia, Philadelphia, PA; 2 Division of Nephrology, The Children's Hospital of Philadelphia, Philadelphia, PA; 3 Department of Biomedical Research, A.I. duPont Hospital for Children, Wilmington, DE; 4 Department of Medical Imaging, A.I. duPont Hospital for Children, Wilmington, DE; 5 Department of Orthopedics, University of North Carolina at Chapel Hill, Chapel Hill, NC

**Keywords:** BMD, distal femur, DXA, children, bone densitometry, reference data

## Abstract

**Background:**

Lateral distal femur (LDF) scans by dual energy x-ray absorptiometry (DXA) are often feasible in children for whom other sites are not measurable. Pediatric reference data for LDF are not available for more recent DXA technology.

**Aims:**

To assess older pediatric LDF reference data, construct new reference curves for LDF bone mineral density (BMD), and demonstrate the comparability of LDF BMD to other measures of BMD and strength assessed by DXA and by peripheral quantitative computed tomography (pQCT).

**Methods:**

LDF, spine and whole body scans of 821 healthy children, 5 to 18 years of age, recruited at a single center were obtained using a Hologic Delphi/Discovery system. Tibia trabecular and total BMD (3% site), cortical geometry (38% site) (cortical thickness, section modulus, strain strength index) were assessed by pQCT. Sex and race-specific reference curves were generated using LMS-ChartMaker and Z-scores calculated and compared by correlation analysis.

**Results:**

Z-scores for LDF BMD based on published findings demonstrated overestimation or underestimation of the prevalence of low BMD-for-age depending on the region of interest considered. Revised LDF reference curves were generated. The new LDF Z-scores were strongly and significantly associated with weight, BMI, spine and whole body BMD Z-scores, and all pQCT Z-scores.

**Conclusion:**

These findings demonstrate the comparability of LDF measurements to other clinical and research bone density assessment modes, and enable assessment of BMD in children with disabilities, who are particularly prone to low trauma fractures of long bones, and for whom traditional DXA measurement sites are not feasible.

## Introduction

Children with physical disabilities such as cerebral palsy, spina bifida, muscular dystrophy, and spinal cord injuries that limit ambulation are typically osteopenic ^1-3^. This in turn results in fractures with minimal, or in some cases even unrecognized trauma. Femoral shaft and distal metaphyseal fractures are particularly common^4,5^. Assessment of bone density in these conditions is made difficult by several factors. Contractures of the lower limbs are prevalent and prevent laying in a fully supine position for optimal whole body and proximal femur (hip) measurements by dual energy x-ray absorptiometry (DXA). In addition, the anatomy of the proximal femur is frequently distorted in these conditions due to dysplasia, subluxation, or hip dislocation. Clinical care of hip disorders in these conditions sometimes requires osteotomy procedures and internal fixation with metallic implants, further interfering with DXA bone density assessment in this region.

Bone density measurement in the lumbar spine is also problematic in children with many common physical disabilities. The anatomy is often distorted due to scoliosis, which if surgically treated will have metallic fixation that interferes with DXA imaging. An additional point regarding bone density measurements in the lumbar spine is the lack of relevance to fracture risk in this population A prospective, longitudinal study in children with quadriplegic cerebral palsy found that DXA measures of lumbar spine areal bone mineral density (aBMD) were not predictive of subsequent fracture risk ^6^. This somewhat surprising observation likely relates to the finding that aBMD measures in the femur and spine correlate poorly in a child with low BMD ^7^. Fractures in children with physical disabilities typically occur in the long bones, most commonly the femur and tibia ^8,9^. In marked distinction to elderly adults, osteoporotic compression fractures of the spine are uncommon in nonambulatory children.

In order to address these difficulties in obtaining clinically meaningful assessments of bone health in children with disabilities an alternative technique was developed utilizing DXA measurements of the distal femur projected in the lateral plane^10,11^. Advantages of this technique are that the femur is the most common site of fracture, children with severe contractures can be comfortably positioned, and metallic fixation is rarely utilized in this region. Further, subregional analyses allow separate assessment of regions rich in cortical versus cancellous bone.

Existing reference data for bone density of the distal femur is based upon a relatively small sample of healthy children who were measured with the older pencil beam DXA technology ^11^. The purpose of this report is to provide more robust pediatric DXA lateral distal femur (LDF) aBMD reference data utilizing contemporary fan-beam technology. These LDF reference data were compared to DXA measures of areal BMC and aBMD of the spine and whole body, the sites recommended for clinical assessment of bone density in children ^12^. In addition, LDF bone density was compared to tibia measures of trabecular and cortical volumetric BMD (vBMD) and geometry measured by peripheral quantitative computed tomography (pQCT). Unlike DXA aBMD measures, which are based on a two-dimensional bone image, pQCT provides a three-dimensional vBMD measure, distinct estimates of trabecular and cortical vBMD, and measures of bone geometry known to relate to bone strength^13^. These comparisons were performed as a relative validation of the LDF measurement with respect to other commonly used clinical and research methods for bone density assessment.

## Methods

### Sample

Study participants consisted of healthy children, 5 to 18 years of age, enrolled in the Reference Project on Skeletal Development at The Children's Hospital of Philadelphia. Subjects were recruited through the pediatric practices of The Children's Hospital of Philadelphia, newspaper advertisements and community fliers. Children were excluded for chronic health conditions (e.g., renal, endocrine, gastrointestinal disorders) and medication use (e.g., glucocorticoids) that might affect growth or development (premature birth), dietary intake (medications affecting appetite), or bone density (restricted ambulation). Children were not excluded on the basis of fracture history, since fractures occur in 25 to 50% of otherwise healthy children^14,15^. Parents or guardians of subjects less than 18 years of age provided written informed consent, and the subjects provided assent. Subjects 18 years of age provided informed consent. The protocol was approved by the Internal Review Board of The Children's Hospital of Philadelphia.

### Assessment of Growth and Pubertal Development

Height was measured to the nearest 0.1cm with a stadiometer (Holtain, Crymych, UK) and weight to the nearest 0.1kg a digital scale (Scaletronix). All measurements were obtained in triplicate by a trained anthropometrist using standardized techniques ^16^ with the subject wearing light clothing and with shoes and hair adornments removed. The mean of the three measurements was used in the analysis. The stage of pubertal development was determined using a validated self-assessment questionnaire ^17,18^ and classified according to Tanner ^19^.

### DXA Measures of Bone Density

Areal bone mineral density (aBMD) measurements were obtained by dual energy x-ray absorptiometry (DXA) with a Delphi/Discovery (Hologic, Bedford, MA) densitometer. All measurements were obtained with the same device and analyzed using software version 12.3. The DXA exam included scans of the lumbar spine and whole body following standardized positioning, acquisition and analysis techniques. The coefficient of variation (%CV) for DXA measurements of the spine and whole body in children range from 0.64 to 1.20 ^20^.

**Figure 1:**
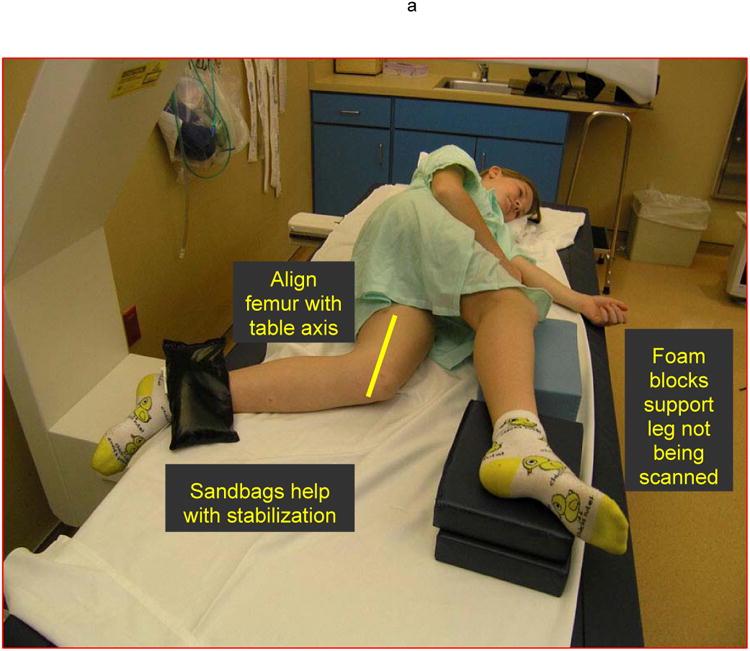

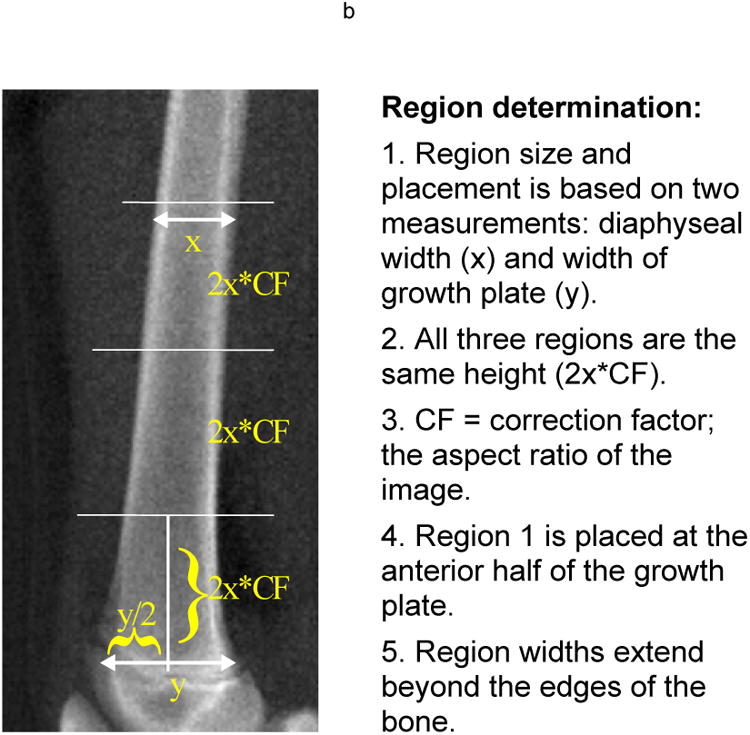

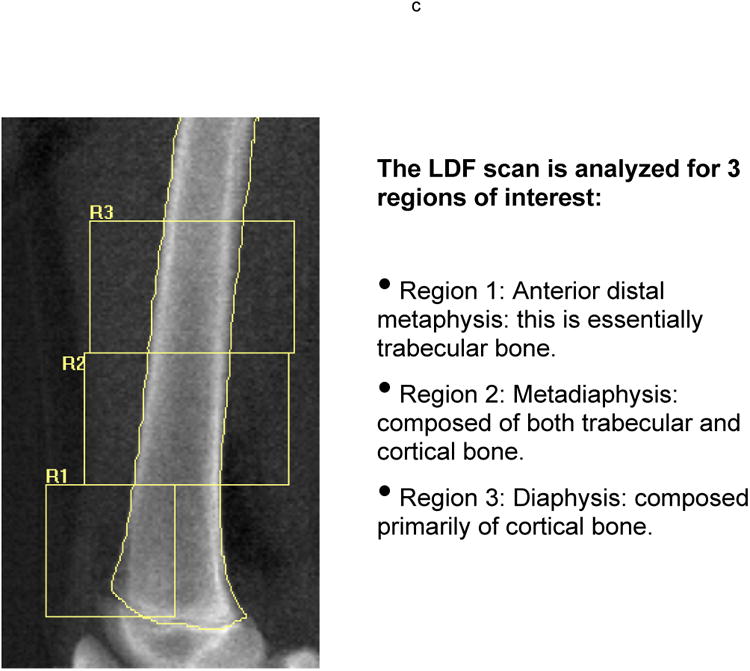
(A) Patient positioning for the left lateral distal femur scan showing the child in a side-lying position with positioning devices (foam blocks and sand bags) to assist in attaining a comfortable and stable position. The femur is centered on the table and parallel to the edge. The forearm scan mode is used to obtain the scan. (B) Analysis of the scan requires insertion of region of interest boxes. The width and height of each region of interest box is illustrated in the figure. (C) The three regions of interest are illustrated in the figure.

The lateral distal femur scan was obtained as described in Henderson et al. ^21^; positioning of the subject is illustrated in Figure 1a, and placement of the regions of interest (ROIs) is shown in Figures 1b and 1c. Briefly, the patient is placed in a side lying position on the scanning table, on the side being measured, with the femur following the length of the table. The other thigh is flexed out of the field of view. The lateral distal femur is analyzed for three regions of interest: region 1 is placed at the anterior half of distal metaphysis, region two is metadiaphyseal, and region 3 is diaphyseal. Region size is based on the diaphyseal width; all three regions are the same height. To assure consistency in lateral distal femur scan acquisition and analysis techniques, a subset of 40 randomly selected scans were reviewed by an independent investigator (HK). In addition, all scans were inspected by one investigator (BZ) for movement, interfering factors, and analysis consistency (placement of regions of interest) to assure technical quality of all scans.

### pQCT Measures of Volumetric BMD and Bone Strength

Volumetric BMD (vBMD) and bone strength of the distal tibia were assessed by peripheral quantitative computed tomography (pQCT) in a subset of the healthy reference sample using a Stratec XCT2000 device (Orthometrix, White Plains, NY) with a voxel size of 0.4mm and scan speed of 25mm/sec. An anthropometric measure of tibia length (mm) (from the distal tip of the medial malleolus to the superior edge of the medial tibial plateau) was obtained using sliding calipers (Rosscraft, Vancouver, BC). A scout view was obtained to place the reference line at the proximal border of the distal tibia growth plate, and measurements were obtained at regions located at the distal 3% and 38% of tibia length. Scans were analyzed using the Stratec 5.50 software. At the 3% site, scans were analyzed for total and trabecular vBMD^1^. At the 38% site, scans were analyzed for cortical thickness, section modulus and the strain strength index (SSI)^2^. The coefficient of variation (%CV) for selected pQCT measures were as follows: trabecular density 1.4%, cortical thickness 1.4%, SSI 2.8% ^22^.

### Statistical Analysis

Descriptive statistics and graphical displays were generated to characterize the data and assess the distributions. Height, weight and body mass index (BMI) were converted to Z-scores (standard deviation scores) based on the Centers for Disease Control and Prevention Growth Charts ^23^.

Z-scores for lateral distal femur aBMD were calculated based on previously published reference data for lateral distal femur aBMD^11^. For males and females separately, the student's T-test was used to determine whether the distribution of Z-scores was significantly different than expected for a healthy reference samples, i.e., a mean of zero and standard deviation of one.

To assess the need for sex and race-specific reference curves, multiple linear regression analyses were used to determine whether significant group differences existed. Subjects were categorized as black vs. non-black according to self-reported race. Based on these analyses (results not presented), it was determined that separate sex- and race-specific reference curves were needed.

Reference curves for lateral distal femur aBMD measurements were generated using the “LMS” method ^24^ that accounts for the non-linearity, heteroscedasticity and skewness of aBMD data in growing children. Sex and race specific curves were constructed for aBMD of regions 1 through 3 using the LMS Chartmaker Program version 2.3^25^. The LMS method fits three parameters (LMS) as cubic splines by nonlinear regression. The three parameters represent the median (M), standard deviation (S), and power in the Box-Cox transformation (L) that vary as a function of age. These parameters are used to construct centile curves using the following formula:

Equation 1 $$\text{BMD centile}=M{\left(1+L\cdot S\cdot Z\right)}^{1/L}$$

where the L, M, and S are age-specific, and the Z is the Z-score that corresponds to a given percentile (e.g., Z=0 is the 50^th^ percentile). For an individual with a measurement X, a Z-score is calculated using the age-specific L, M and S parameters and the following formula:

Equation 2 $$Z=[{\left(X/M\right)}^{L})-1]/L\cdot S$$

Fit of the curves was evaluated by graphical inspection of the centile curves relative to the raw data and by Q-Q plots.

The LMS method was also used to generate sex and race-specific reference curves for all other densitometric measures on the same sample of subjects. Z-scores for DXA measures of spine aBMC and aBMD, total body aBMC and aBMD, and pQCT measures of trabecular and total vBMD at the 3% site of the tibia relative to age, and for cortical thickness, section modulus and strain-strength index relative to tibia length were calculated. Additional statistical analyses included correlations among the Z-scores for growth, lumbar spine, whole body and lateral distal femur aBMD, and pQCT measures of vBMD and bone strength.

## Results

Lateral distal femur results were available for 821 of the 854 subjects recruited. The subjects for who lateral distal femur BMD scans were excluded for technical limitations (less than 4%) did not differ in age, sex, race or spine aBMD Z-score from the remainder of the sample. Characteristics of the sample are shown in Table 1. Similar to other reports of U.S. children^26,27^, Z-scores for height, weight and BMI were above zero signaling that their growth differed somewhat from the CDC Growth Charts. This sample was similar in height Z-score, but had lower weight and BMI Z-scores than the sample used in the development of the original LDF reference data (0.6±1.0 for both Z-scores) ^11^. Descriptive statistics (mean and standard deviation) for all bone measures are given in Table 2. The study subjects had DXA bone density values that were similar to U.S. national reference data^26^ (available only for ages 7 to 17: spine aBMD Z-score: -0.07±1.0, n=612; whole body aBMD Z-score: 0.09±1.0, n=668). Due to the limitations of the scan field length relative to femur dimensions, fewer subjects had results for lateral distal femur Region 3 (n=625) compared to Region 1 (n=821).

Z-scores for aBMD of the lateral distal femur based on previously published reference data^11^ acquired with a pencil-beam device are shown in Table 3. All Z-scores were significantly different from zero, and varied by sex, race and site. Thus, use of this reference data may lead to overestimation of the prevalence of low BMD-for-age (defined by the International Society for Clinical Densitometry^12^ as Z-score < -2.0) in some groups, and overestimation in other groups, depending on the region of interest considered.

**Figure 2:**
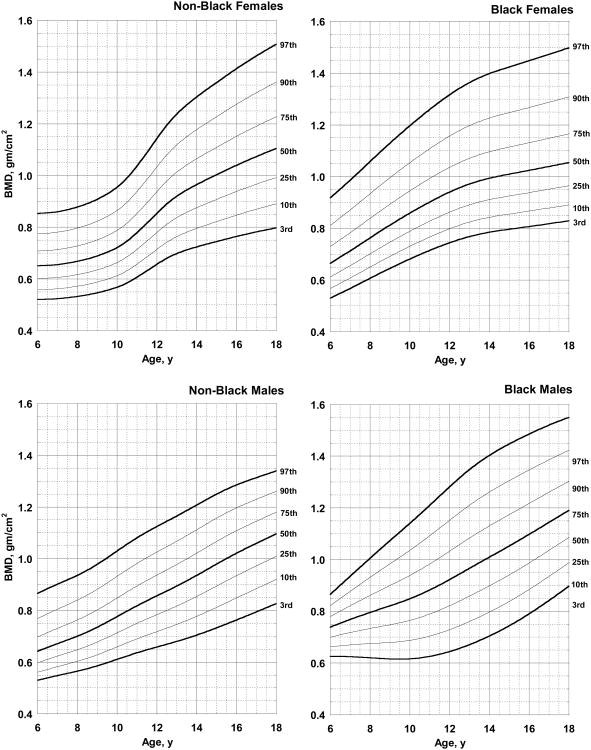
Reference curves for areal bone mineral density for Region 1. The reference curves are based on 244 non-black females, 183 black females, 212 non-black males and 182 black males.

**Figure 3:**
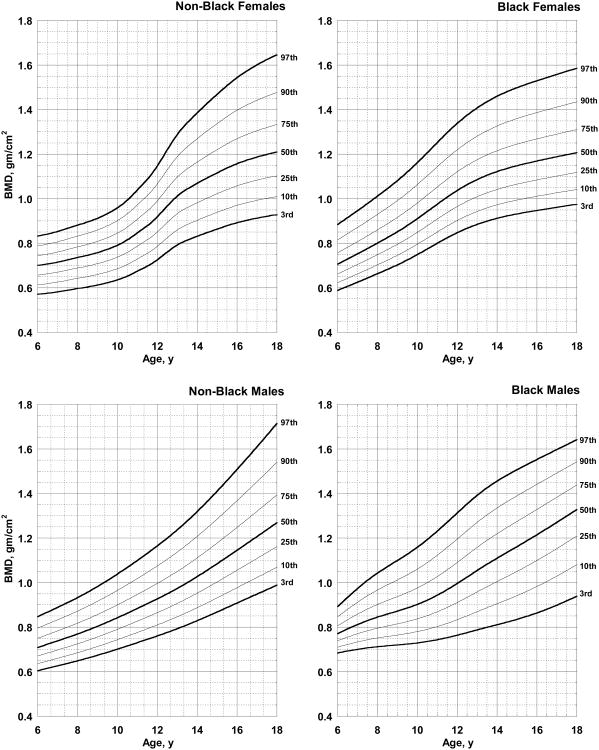
Reference curves for areal bone mineral density for Region 2. The reference curves are based on 244 non-black females, 183 black females, 211 non-black males and 180 black males.

**Figure 4:**
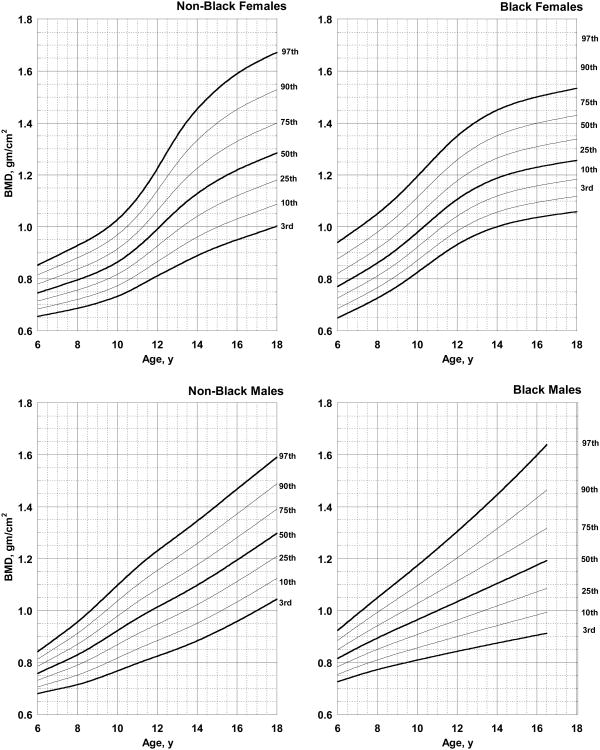
Reference curves for areal bone mineral density for Region 3. The reference curves are based on 184 non-black females, 146 black females, 157 non-black males and 138 black males. The curve for black males is restricted to ages 6 to 16 because there was insufficient representation of subjects in this age range.

The new reference curves for lateral distal femur aBMD resulted in Z-scores with a mean of zero and standard deviation of one, as expected. The L, M, and S parameters and percentile distributions are given in Table 4a to 4c, and the distributions of lateral distal femur aBMD are shown in Figures 2 to 4. Because of the small number of subjects in the 5 year old age group (n=13, divided by race and sex), the values in Table 4a to 4c were restricted to ages 6 to 18. The L, M (50^th^ percentile) and S values in Table 4 can be used to calculate a Z-score for an individual child using equations 1 and 2 given above.

DXA spine and whole body scans were available for 838 subjects, and pQCT scans were available for 566 subjects at the 3% site and 610 subjects at the 38% site. Since values for these measures increased with age and body size in a non-linear fashion, Z-scores were computed for all measures to account for these expected changes. The mean ± sd was 0±1 for each of these Z-scores, e.g., aBMD of the spine and whole body and pQCT measures of vBMD and bone strength, as expected. Correlations among Z-scores for growth/body size (height, weight, BMI), aBMD and vBMD and strength measures are shown in Table 5. These correlation coefficients illustrate that lateral distal femur aBMD Z-scores were significantly associated with other DXA measures of aBMD recommended for clinical assessment in children^12^. The lateral distal femur Z-scores, like other aBMD Z-scores, were positively and significantly associated with growth (height, weight and BMI) Z-scores indicating that children who were large for their age had greater aBMC and aBMD for their age. Also of note, lateral distal femur Z-scores, like the spine and whole body Z-scores, correlated well with pQCT measures of vBMD and bone strength, especially the measures of trabecular and total vBMD.

## Discussion

For many children with disabilities and at-risk for the co-morbidity of low BMD, traditional assessment techniques such as spine or whole body imaging by DXA are often not an option due to deformity, indwelling hardware or difficulties with positioning. In addition, lower limb fractures are particularly common among children with conditions affecting mobility, such as cerebral palsy and Duchenne muscular dystrophy ^4,5^, so the distal femur is of significant clinical interest. Distal femur scans offer an excellent alternative for most of these children. Previously published reference data for the lateral distal femur were based on a smaller sample of 256 healthy children, primarily Caucasian ^11^. These scans were obtained on older technology, the Hologic (QDR1000 and 2000 models) scanners in pencil-beam mode. The current generation of fan-beam technology DXA instruments offer more rapid scan times; a factor that is critical in the success of obtaining interpretable scans in children, especially those with significant physical or cognitive disabilities. In addition, changes in DXA instruments and software are known to have a significant impact on aBMD results in children because of their smaller bone size and soft tissue distribution ^28,29^. The results of this study demonstrated that the distribution of lateral distal femur aBMD in this large sample of healthy children measured in the latest generation of Hologic software and hardware in fan-beam mode differed from the previously published data. Thus, when selecting reference data for the evaluation of measurements in children, it is important to consider the hardware and software versions used^12^.

Optimally, pediatric reference data should be based on a sample of healthy children that is sufficiently large to characterize the age and sex related variability in the outcome measure, and on statistical techniques that accurately characterize the variability in the measure. Racial differences in aBMD during childhood add further complexity to the development of reference data. For most BMD measures, the age-related changes are non-linear and the variability increases with age. The reference ranges presented here were based on a total of 821 children, with at least 180 children in each sex and race group. The statistical technique used to generate the reference ranges was the same as that used for the creation of the CDC growth charts^23^ and the U.S. reference data for aBMD^26^. This approach enables calculation of exact centiles and Z-scores using equations 1 and 2 (above) and the L, M, and S values from Table 4. A possible limitation of the reference data presented is that all subjects were evaluated at a single geographical location drawing on subjects from urban and suburban communities. Potential regional and device-specific variation in lateral distal femur aBMD could not be assessed. In addition, because of limitations in the length of the scan field relative to the size of the femur, not all regions of interest could be measured in all subjects.

Separate reference curves were presented for Black vs. Non-Black children. Racial differences in BMD are widely recognized in adults and children ^30-34^. However, the clinical application of race-specific reference curves should be carefully considered. The International Society for Clinical Densitometry recommends the use of reference curves based on Caucasian samples for adults for all race and ethnic groups ^35^. For children, the use of race-specific curves is recommended^12^ since they may be useful in identifying children who are not attaining their genetic potential for bone mineral accrual. However, the use of race-specific reference curves for determination of fracture risk in children is unknown and requires further investigation.

Z-scores for lateral distal femur aBMD were compared to Z-scores for growth, traditional DXA measures of aBMD, and pQCT measures of vBMD and bone strength. While none of these comparisons attest to the accuracy of lateral distal femur aBMD measurements since they are measuring different aspects of skeletal and somatic growth and development, they do provide important insights. First, the lateral distal femur aBMD Z-scores were significantly associated with body size measures, as were other aBMD Z-scores. In other words, children who were large for their age also had higher aBMD for their age. Of note, the association between height Z-score and lateral distal femur aBMD Z-score was somewhat lower than the corresponding associations between height Z-score and spine and whole body aBMD Z-score. This is likely due to the technique used in the analysis of the lateral distal femur image, in which bone size is taken into account in defining the region of interest. This is not done in spine or whole body DXA measurements. Since many children at-risk for low BMD also have growth failure, it is an important consideration that this measure of aBMD status is less influenced by height status. It is also noteworthy that the correlations between lateral distal femur aBMD and weight and BMI Z-score were higher than for other aBMD measures. One possible explanation is that the distribution of weight-bearing forces are more concentrated on the lateral distal femur than on the spine or whole body, so the strength of this association may reflect the impact of weight-bearing physical activity on BMD of the femur.

Lateral distal femur aBMD Z-scores were also strongly associated with Z-scores of distal tibia total and trabecular vBMD, and mid-shaft cortical thickness, section modulus and SSI obtained by pQCT. These pQCT measures offer more detail than DXA various aspects of bone strength (trabecular bone at the ultradistal 3% site and cortical bone in the mid-shaft at the 38% site) and therefore provide further evidence of the utility and validity of the lateral distal femur scan to characterize bone strength. The use of Z-scores in the analysis has the advantage of removing the age or size effects associate with the unadjusted bone measurements. Z-scores are an indicator of status relative to peers of the same age and sex. The correlations among Z-scores demonstrated, for example, the degree to which a healthy child who has a high lateral distal femur aBMD for age will also have a high trabecular vBMD relative to same age peers. The correlation results also demonstrated that the lateral distal femur aBMD Z-scores performed as well or better than spine and whole body Z-scores in relation to pQCT measures of trabecular and cortical bone in healthy children.

Unlike DXA measures of aBMD, pQCT vBMD Z-scores were not influenced by height status ^36^. Although these pQCT measures were obtained on the tibia rather than the femur (due to the configuration of the pQCT device), the significant associations between the lateral distal femur aBMD Z-scores and pQCT outcome measures indicates the degree to which lateral distal femur DXA aBMD measurements are generalizable to other measures obtained with a method that is strongly predictive of fracture risk in experimental situations^37,38^.

In summary, reference curves for lateral distal femur aBMD measurements to be used for clinical care are presented based on a large multi-ethnic sample of healthy children. This technique provides an excellent opportunity for bone health assessment in children for whom spine or whole body measurements are not feasible. A critical issue for any measure of bone health is the relationship to fracture. Futures studies are needed to evaluate the ability of lateral distal femur aBMD measurements to predict fracture, especially among children for whom traditional DXA measurement sites are not feasible.

## Figures and Tables

**Table 1:** Sample Characteristics (n=821)

Gender	52% Female
Race	45% Black / African American
Sexual Maturity	
Tanner 1	34%
Tanner 2	14%
Tanner 3	15%
Tanner 4	23%
Tanner 5	15%
Age, y*	11.4 (3.5)
Height Z-score*	0.3 (0.9)
Weight Z-score*	0.4 (1.0)
BMI Z-score*	0.3 (1.0)

* mean (sd)

**Table 2:** Bone Density Measures by DXA and pQCT

Measure	n	Mean	s.d.
**Lateral Distal Femur**			

Region1 aBMD, gm/cm^2^	821	0.88	0.19
Region2 aBMD, gm/cm^2^	818	0.96	0.21
Region3 aBMD, gm/cm^2^	625	0.93	0.23

**Lumbar Spine and Whole Body DXA**			

Lumbar Spine aBMC, gm	810	35.1	17.0
Lumbar Spine aBMD, gm/cm^2^	810	0.73	0.19
Whole Body aBMC, gm	814	1393	554

**Tibia pQCT**			

Tibia Total vBMD (3% site), gm/cm^3^	546	307	39
Tibia Trabecular vBMD (3% site), gm/cm^3^	546	248	31
Tibia Cortical Thickness, mm	587	4.47	0.90
Tibia Section Modulus, mm^3^	587	1210	537
Tibia Strain Strength Index, mm^4^	587	1128	508

aBMD: areal bone mineral density

vBMD: volumetric bone mineral density

**Table 3:** Lateral Distal Femur BMD Z-Scores (Regions 1 and 2) For The Current Sample Based On Previously Published Reference Data^11^

Race	Gender	Region 1 BMD Z-score			Region 2 BMD Z-score		
	
		Mean	Sd	p	Mean	sd	P
All	All	-0.13	1.11	<0.001	0.52	1.07	<0.001
Non-Black	Females	-0.46	1.09	<0.001	0.21	1.07	0.003
	Males	-0.39	0.82	<0.001	0.25	0.82	<0.001
Black	Females	0.25	1.31	0.01	0.94	1.16	<0.001
	Males	0.23	1.02	0.003	0.84	1.01	<0.001

p-value based on a t-test to determine if the mean is significantly different from zero.

**Table 4:** 

a. L, M, S parameters and percentile distributions for Region 1 BMD														
	NON BLACK													
	
	Females							Males						
	
Age	L	S	3rd	10th	M 50th	90th	97th	L	S	3rd	10th	M 50th	90th	97^th^
	
6.0	-0.78	0.12	0.52	0.56	0.65	0.78	0.85	-1.83	0.12	0.53	0.56	0.64	0.77	0.87
7.0	-0.77	0.12	0.52	0.56	0.66	0.78	0.86	-1.51	0.12	0.55	0.58	0.67	0.80	0.90
8.0	-0.74	0.12	0.53	0.57	0.67	0.80	0.88	-1.20	0.12	0.57	0.60	0.70	0.84	0.94
9.0	-0.69	0.13	0.55	0.59	0.69	0.82	0.91	-0.90	0.13	0.59	0.63	0.73	0.88	0.98
10.0	-0.61	0.13	0.57	0.61	0.72	0.86	0.95	-0.64	0.13	0.61	0.66	0.78	0.93	1.03
11.0	-0.51	0.13	0.61	0.66	0.78	0.94	1.04	-0.39	0.13	0.64	0.69	0.82	0.98	1.08
12.0	-0.38	0.14	0.66	0.71	0.85	1.03	1.14	-0.13	0.13	0.66	0.72	0.86	1.03	1.12
13.0	-0.26	0.14	0.70	0.76	0.92	1.12	1.24	0.12	0.13	0.68	0.75	0.89	1.07	1.17
14.0	-0.16	0.15	0.72	0.80	0.96	1.18	1.30	0.38	0.13	0.70	0.78	0.94	1.11	1.21
15.0	-0.07	0.15	0.74	0.82	1.00	1.23	1.36	0.65	0.13	0.73	0.81	0.98	1.16	1.25
16.0	0.00	0.15	0.76	0.85	1.04	1.28	1.41	0.91	0.13	0.76	0.85	1.02	1.20	1.29
17.0	0.08	0.16	0.78	0.87	1.07	1.32	1.46	1.17	0.12	0.79	0.88	1.06	1.23	1.31
18.0	0.14	0.16	0.80	0.89	1.10	1.36	1.51	1.43	0.12	0.83	0.92	1.10	1.26	1.34
	
	**BLACK**													
	
	**Females**							**Males**						
	
age	L	S	3rd	10th	M 50th	90th	97th	L	S	3rd	10th	M 50th	90th	97^th^
	
6.0	-1.29	0.13	0.53	0.57	0.66	0.81	0.92	0.26	0.08	0.63	0.70	0.78	0.82	0.87
7.0	-1.29	0.13	0.57	0.61	0.71	0.87	0.99	0.26	0.10	0.62	0.72	0.82	0.88	0.94
8.0	-1.29	0.13	0.61	0.65	0.76	0.93	1.06	0.26	0.12	0.62	0.73	0.86	0.93	1.01
9.0	-1.29	0.13	0.64	0.69	0.81	1.00	1.13	0.26	0.14	0.62	0.75	0.90	0.98	1.07
10.0	-1.29	0.13	0.68	0.73	0.86	1.05	1.20	0.26	0.15	0.62	0.76	0.94	1.04	1.14
11.0	-1.29	0.14	0.71	0.77	0.90	1.11	1.26	0.26	0.16	0.63	0.79	0.98	1.09	1.21
12.0	-1.29	0.14	0.74	0.80	0.94	1.16	1.32	0.26	0.17	0.64	0.82	1.03	1.15	1.28
13.0	-1.29	0.14	0.77	0.82	0.97	1.20	1.36	0.26	0.17	0.67	0.86	1.08	1.21	1.35
14.0	-1.29	0.14	0.78	0.84	0.99	1.23	1.40	0.26	0.17	0.70	0.90	1.13	1.26	1.40
15.0	-1.29	0.14	0.80	0.86	1.01	1.25	1.42	0.26	0.17	0.74	0.94	1.17	1.31	1.45
16.0	-1.29	0.14	0.81	0.87	1.02	1.27	1.45	0.26	0.16	0.79	0.99	1.22	1.35	1.49
17.0	-1.29	0.14	0.82	0.88	1.04	1.29	1.47	0.26	0.15	0.84	1.04	1.26	1.39	1.52
18.0	-1.29	0.14	0.83	0.89	1.05	1.31	1.50	0.26	0.14	0.90	1.09	1.30	1.42	1.55

b. L, M, S parameters and percentile distributions for Region 2 BMD														
	NON BLACK													
	
	Females							Males						
	
Age	L	S	3rd	10th	M 50th	90th	97th	L	S	3rd	10th	M 50th	90th	97^th^
	
6.0	0.88	0.09	0.57	0.61	0.70	0.79	0.83	-0.68	0.08	0.60	0.64	0.71	0.80	0.85
7.0	0.84	0.09	0.58	0.63	0.72	0.81	0.85	-0.68	0.09	0.63	0.66	0.74	0.83	0.89
8.0	0.78	0.10	0.60	0.64	0.74	0.83	0.88	-0.68	0.09	0.65	0.69	0.77	0.87	0.93
9.0	0.72	0.10	0.61	0.66	0.76	0.86	0.91	-0.68	0.09	0.67	0.71	0.80	0.92	0.98
10.0	0.62	0.10	0.64	0.69	0.79	0.90	0.96	-0.68	0.10	0.70	0.74	0.84	0.97	1.04
11.0	0.47	0.11	0.67	0.73	0.85	0.97	1.04	-0.68	0.10	0.73	0.78	0.88	1.02	1.10
12.0	0.27	0.11	0.73	0.79	0.92	1.07	1.15	-0.68	0.11	0.76	0.81	0.93	1.07	1.16
13.0	0.06	0.12	0.79	0.86	1.01	1.19	1.29	-0.68	0.11	0.79	0.85	0.97	1.14	1.24
14.0	-0.12	0.13	0.83	0.90	1.07	1.27	1.39	-0.68	0.12	0.83	0.89	1.03	1.21	1.32
15.0	-0.26	0.13	0.86	0.94	1.12	1.34	1.47	-0.68	0.12	0.87	0.93	1.08	1.29	1.41
16.0	-0.38	0.14	0.89	0.97	1.16	1.40	1.54	-0.68	0.13	0.91	0.98	1.15	1.37	1.51
17.0	-0.47	0.14	0.91	0.99	1.19	1.44	1.60	-0.68	0.13	0.95	1.02	1.21	1.45	1.61
18.0	-0.54	0.14	0.93	1.01	1.21	1.48	1.65	-0.68	0.14	0.99	1.07	1.27	1.54	1.72
	
	**BLACK**													
	
	**Females**							**Males**						
	
age	L	S	3rd	10th	M 50th	90th	97th	L	S	3rd	10th	M 50th	90th	97th
	
6.0	-1.03	0.10	0.59	0.62	0.71	0.82	0.88	-1.53	0.07	0.68	0.71	0.77	0.85	0.89
7.0	-1.03	0.10	0.62	0.66	0.75	0.87	0.95	-1.29	0.08	0.70	0.73	0.81	0.91	0.97
8.0	-1.03	0.10	0.66	0.70	0.80	0.93	1.01	-1.08	0.09	0.71	0.75	0.84	0.97	1.04
9.0	-1.03	0.11	0.70	0.75	0.85	0.99	1.08	-0.89	0.10	0.72	0.76	0.87	1.01	1.10
10.0	-1.03	0.11	0.75	0.80	0.91	1.06	1.16	-0.69	0.11	0.73	0.78	0.90	1.06	1.16
11.0	-1.03	0.11	0.80	0.85	0.98	1.14	1.25	-0.44	0.13	0.74	0.80	0.94	1.12	1.23
12.0	-1.03	0.11	0.85	0.90	1.04	1.22	1.34	-0.15	0.14	0.76	0.83	1.00	1.20	1.31
13.0	-1.03	0.11	0.88	0.94	1.09	1.28	1.41	0.17	0.14	0.79	0.87	1.05	1.27	1.39
14.0	-1.03	0.12	0.91	0.97	1.12	1.33	1.46	0.49	0.15	0.81	0.91	1.11	1.34	1.46
15.0	-1.03	0.12	0.93	0.99	1.15	1.36	1.50	0.81	0.14	0.83	0.94	1.16	1.39	1.51
16.0	-1.03	0.12	0.95	1.01	1.17	1.39	1.53	1.13	0.14	0.86	0.98	1.21	1.44	1.55
17.0	-1.03	0.12	0.96	1.03	1.19	1.41	1.56	1.46	0.14	0.90	1.03	1.27	1.49	1.60
18.0	-1.03	0.12	0.98	1.04	1.21	1.44	1.59	1.79	0.13	0.94	1.08	1.33	1.54	1.64

c. L, M, S parameters and percentile distributions for Region 3 BMD														
	NON BLACK													
	
	Females							Males						
	
Age	L	S	3rd	10th	M 50th	90th	97th	L	S	3rd	10th	M 50th	90th	97th
	
6.0	-0.25	0.07	0.66	0.68	0.75	0.82	0.85	0.32	0.05	0.68	0.71	0.76	0.81	0.84
7.0	-0.25	0.07	0.67	0.70	0.77	0.85	0.89	0.32	0.06	0.70	0.73	0.79	0.86	0.90
8.0	-0.25	0.08	0.69	0.72	0.80	0.88	0.93	0.32	0.07	0.71	0.75	0.83	0.91	0.96
9.0	-0.25	0.08	0.71	0.74	0.83	0.92	0.97	0.32	0.08	0.74	0.78	0.87	0.97	1.02
10.0	-0.25	0.08	0.73	0.77	0.86	0.97	1.03	0.32	0.09	0.77	0.82	0.92	1.04	1.10
11.0	-0.25	0.09	0.77	0.82	0.92	1.04	1.11	0.32	0.10	0.80	0.85	0.97	1.10	1.17
12.0	-0.25	0.10	0.81	0.87	0.99	1.14	1.22	0.32	0.10	0.82	0.88	1.01	1.15	1.23
13.0	-0.25	0.12	0.85	0.92	1.07	1.25	1.35	0.32	0.10	0.85	0.92	1.05	1.21	1.29
14.0	-0.25	0.12	0.89	0.96	1.13	1.33	1.45	0.32	0.10	0.88	0.95	1.10	1.26	1.34
15.0	-0.25	0.13	0.92	1.00	1.18	1.40	1.53	0.32	0.11	0.92	0.99	1.14	1.31	1.41
16.0	-0.25	0.13	0.95	1.03	1.22	1.45	1.59	0.32	0.11	0.96	1.03	1.19	1.37	1.47
17.0	-0.25	0.13	0.98	1.06	1.25	1.49	1.63	0.32	0.11	1.00	1.08	1.25	1.43	1.53
18.0	-0.25	0.13	1.00	1.09	1.28	1.53	1.67	0.32	0.11	1.04	1.12	1.30	1.49	1.59
	
	**BLACK**													
	
	**Female**							**Males**						
	
age	L	S	3rd	10th	M 50th	90th	97th	L	S	3rd	10th	M 50th	90th	97th
	
6.0	-0.82	0.09	0.65	0.69	0.77	0.88	0.94	-0.59	0.06	0.73	0.75	0.82	0.89	0.92
7.0	-0.82	0.09	0.69	0.72	0.81	0.93	0.99	-0.59	0.07	0.75	0.78	0.86	0.94	0.99
8.0	-0.82	0.09	0.73	0.77	0.86	0.98	1.05	-0.59	0.08	0.77	0.81	0.89	0.99	1.05
9.0	-0.82	0.09	0.77	0.81	0.92	1.04	1.12	-0.59	0.08	0.79	0.83	0.93	1.04	1.11
10.0	-0.82	0.09	0.82	0.87	0.98	1.11	1.19	-0.59	0.09	0.81	0.86	0.96	1.10	1.17
11.0	-0.82	0.09	0.88	0.93	1.05	1.19	1.28	-0.59	0.10	0.83	0.88	1.00	1.15	1.24
12.0	-0.82	0.09	0.93	0.98	1.11	1.26	1.35	-0.59	0.11	0.84	0.90	1.03	1.20	1.30
13.0	-0.82	0.09	0.97	1.03	1.15	1.31	1.41	-0.59	0.12	0.86	0.92	1.07	1.26	1.37
14.0	-0.82	0.09	1.00	1.06	1.19	1.35	1.45	-0.59	0.12	0.87	0.94	1.10	1.32	1.45
15.0	-0.82	0.09	1.02	1.08	1.21	1.38	1.48	-0.59	0.13	0.89	0.96	1.14	1.37	1.52
16.0	-0.82	0.09	1.04	1.09	1.23	1.40	1.50	-0.59	0.14	0.91	0.98	1.18	1.43	1.60
17.0*	-0.82	0.09	1.05	1.11	1.24	1.42	1.52							
18.0*	-0.82	0.09	1.06	1.12	1.26	1.43	1.53							

* too few male subjects in this age range

**Table 5:** Correlations Among Z-Scores*

	Region 1 aBMD	Region 2 aBMD	Spine aBMD	WB aBMC
Region 2 aBMD	0.81	1.00	0.63	0.68
Region 3 aBMD	0.72	0.91	0.58	0.65

Height	0.26	0.26	0.32	0.60
Weight	0.54	0.48	0.44	0.64
BMI	0.50	0.44	0.36	0.45

Spine aBMC	0.55	0.61	0.84	0.85
Spine aBMD	0.61	0.63	1.00	0.78
WB aBMC	0.66	0.68	0.78	1.00
WB aBMD	0.68	0.72	0.77	0.87

Trabecular vBMD	0.61	0.64	0.45	0.46
Total vBMD	0.57	0.63	0.46	0.42
Cortical Thickness	0.44	0.47	0.40	0.31
Section Modulus	0.39	0.36	0.35	0.37
Strain Strength Index	0.36	0.34	0.35	0.37

* Note: all correlations are statistically significant p<0.0001

## Footnotes

1 pQCT analysis parameters used: contour mode 1, peel mode 1, inner threshold=200, outer threshold=600.

2 pQCT analysis parameters used: contour mode 1, peel mode 2, cortmode 2, threshold = 711.For the SSI measure only, the threshold was set to 300.
